# Venous thromboembolism in Latin America: a review and guide to diagnosis and treatment for primary care

**DOI:** 10.6061/clinics/2016(01)07

**Published:** 2016-01

**Authors:** Jose Manuel Ceresetto

**Affiliations:** Hospital Británico, Servicio de Hematología, Solís, Buenos Aires, Argentina

**Keywords:** Venous Thromboembolism, Pulmonary Embolism, Deep Vein Thrombosis, Latin America, Primary Care

## Abstract

There are various region-specific challenges to the diagnosis and effective treatment of venous thromboembolism in Latin America. Clear guidance for physicians and patient education could improve adherence to existing guidelines. This review examines available information on the burden of pulmonary embolism and deep vein thrombosis in Latin America and the regional issues surrounding the diagnosis and treatment of pulmonary embolism and deep vein thrombosis. Potential barriers to appropriate care, as well as treatment options and limitations on their use, are discussed. Finally, an algorithmic approach to the diagnosis and treatment of venous thromboembolism in ambulatory patients is proposed and care pathways for patients with pulmonary embolism and deep vein thrombosis are outlined for primary care providers in Latin America.

## INTRODUCTION

Although multiple studies have evaluated the epidemiology of venous thromboembolism (VTE) in European and American populations, there is limited evidence on the prevalence of VTE and the burden of disease in Latin America. Evidence from a study in the United States suggests that there are differences in the incidence of VTE among white, black, Hispanic, and Asian populations 1. Thus, it may not be appropriate to simply extrapolate the prevalence of VTE in Latin America from data obtained from European and U.S. populations. In the ENDORSE II study 2, approximately 50% of hospitalized patients from across 43 hospitals in Mexico were identified as being at risk of deep vein thrombosis (DVT) or pulmonary embolism (PE) and in a Brazilian study across 3 hospitals, a similar proportion of hospitalized patients were considered to be at high risk of DVT or PE 3. Data from an Argentinian study 4 estimated an incidence rate of 0.7 per 1,000 person-years for total VTE (0.48 and 0.22 for DVT and PE, respectively) based on the incidence rate observed at a Buenos Aires hospital and extrapolated to the entire Argentinian population. The in-hospital mortality rate from VTE was estimated at 19% in an Argentinian hospital by Mazzei et al. 5 and 14.1% in a Brazilian hospital by Volschan et al. 6. A large autopsy-based analysis in a Brazilian hospital identified PE as the cause of death in 2.5% of all deaths of hospitalized patients 7. Thus, available data indicate a significant disease burden in Latin America in terms of morbidity and mortality as well as cost to the healthcare system.

There are various regional challenges to effective VTE diagnosis and treatment in Latin America. A cross-sectional study of internal medicine practitioners in Mexico revealed that the awareness of risk factors for VTE and recommended methods of diagnosis was low 8. A significant proportion of patients diagnosed with VTE in Latin American countries may not receive appropriate anticoagulation and some patients at risk of VTE do not receive appropriate prophylaxis 5,. A Venezuelan study of characteristics of patients with VTE observed that patients with VTE often present with comorbidities that may complicate treatment decisions 17, and subsequent studies implicated these comorbidities as potentially affecting the decision to anticoagulate 18. In a study in Brazil, only 26% of patients at moderate or high risk of VTE received prophylactic anticoagulation 3. In a study of the adequacy of prophylactic anticoagulation in 28 institutions across Argentina 19, surgical patients were more likely to receive adequate prophylaxis than medical patients (71% vs. 63%). By contrast, the Epidemiologic International Day for the Evaluation of Patients at Risk for Venous Thromboembolism in the Acute Hospital Care Setting (ENDORSE) study 20 observed that medical patients were more likely than surgical patients to receive adequate prophylactic anticoagulation in Mexico, Venezuela, Colombia and Brazil. This finding highlights potential differences in educational needs across Latin American regions.

The International Society on Thrombosis and Haemostasis recently performed a worldwide survey as part of the first World Thrombosis Day. Of the Argentinians surveyed, the majority did not recognize the symptoms of DVT or PE 21,22. Globally, concern about thrombosis was second highest in Argentina, but fewer than half of Argentinians surveyed recognized that thrombosis is a preventable disease 21.

Clear guidance and education on how comorbidities may affect therapy might increase healthcare providers' adherence to existing guidelines 23-25. Pilot studies using programs and guidance protocols to facilitate treatment decisions in Brazil and Argentina have demonstrated improvements in the level of appropriate anticoagulation administered to patients with or at risk of VTE 26,27. The aim of this review is to outline an algorithmic approach for primary care providers in Latin America for VTE diagnosis in ambulatory patients and to discuss current and emerging options for the treatment of these patients.

### Venous Thromboembolism: Deep Vein Thrombosis and Pulmonary Embolism

VTE, which includes DVT and PE, is associated with significant morbidity and is a leading cause of cardiovascular death worldwide 28. DVT is a common complication of and the most common cause of rehospitalization following any major surgery. Although less common than DVT, PE is a serious complication that can occur after surgery or in association with cancer, other chronic illnesses and pregnancy 28. Various risk scores have been developed to estimate VTE risk in specific patient populations, such as the Khorana score in patients with cancer 29 and the Caprini score in surgical patients 30.

The Khorana score 29 was developed for use in patients with cancer initiating a new chemotherapy regimen as a simple predictive risk model to identify patients at highest risk for VTE who would most benefit from thromboprophylaxis. The scoring system assigns 2 points for the stomach or pancreas as the primary site of cancer or 1 point each for a primary site in the lungs or genitourinary tract, excluding prostate, lymphoma, or gynecological cancer. An additional point is assigned for each of the following risk factors: pre-chemotherapy platelet count of 350 × 108/L or more; hemoglobin level below 10 g/dL or use of erythrocyte growth factors; a pre-chemotherapy leucocyte count greater than 11 × 109/L; or a BMI of 35 or higher. Patients are categorized as low, medium, or high risk based on their total scores (0, 1-2, and ≥3, respectively).

The Caprini score provides a simple checklist for known VTE risk factors in surgical patients based on a health history scored by the physician. The most recent version 30 incorporates approximately 40 different risk factors with weights of 1 to 5 points each; the total score is used to classify patients as low (scores of 0-1), moderate (scores of 1-2), high (scores of 3-4) and highest (scores of 5 or more) risk, each with a recommended prophylactic regimen.

The Caprini and Khorana scores are recommended by the American College of Chest Physicians guidelines as an objective screening method to identify patients at high risk of VTE who might benefit most from prophylactic anticoagulation 31. However, other specialized VTE risk scores require more extensive clinical validation.

### Deep vein thrombosis

DVT can occur in either the upper or lower extremities. In a recent international study by Lamontagne et al. of 3,746 medical/surgical patients in intensive care, 98% of DVT in this population involved thromboses of the lower extremities 32. Of the remaining events, 72% were upper extremity DVT (UEDVT) 32. Patients who experience VTE are at increased risk of recurrence 33,34, and VTE is often associated with long-term, clinically significant complications, including post-thrombotic syndrome and chronic thromboembolic pulmonary hypertension.

DVT of the lower extremities can be classified as proximal or distal (occurring above or below the popliteal vein, respectively). The former is a more serious condition due to the higher associated risk of thromboembolism. DVT can present as pain or tenderness of the leg, with swelling, erythema, discoloration, or surface vein distension, but also frequently occurs as an asymptomatic condition. The presence of symptoms is often insufficient for diagnosis because other conditions can cause similar symptoms. However, when associated with known risk factors, these symptoms can provoke clinical suspicion of DVT warranting further evaluation 35.

UEDVT accounts for 10% or less of all DVT cases and may be associated with local compressive factors or central venous catheter use 36. Clinical outcomes for UEDVT are similar to those for DVT of the lower extremities 37,38, although the recurrence rate is much lower. There are no published randomized, controlled studies of anticoagulation in UEDVT, but observational studies suggest that treatment with anticoagulants is effective 39,40. In the absence of further information, treatment for UEDVT should be the same as for DVT of the lower extremities 31,41.

### Pulmonary embolism

PE is difficult to diagnose based on clinical symptoms alone and may have a broad spectrum of presentations ranging from shortness of breath, tachypnea and syncope to fever, side stitch and hemoptysis.

Shock and hypotension indicate high-risk PE. Although massive PE is characterized by hypotension (systolic blood pressure <90 mm Hg), it can also be associated with syncope and bradycardia and can cause cardiac arrest with sudden death. Although massive PE is associated with a 90-day mortality risk of 52-58%, it accounts for only approximately 5% of all PE events 42,43.

Submassive PE is characterized by the absence of hypotension and the presence of right ventricular dysfunction or myocardial necrosis due to ischemia of the right heart. Tachycardia may suggest right ventricular dysfunction, but has limited specificity. Additional laboratory parameters indicative of myocardial ischemia or an echocardiogram/computed tomography scan revealing an enlarged right ventricle are also required to confirm a diagnosis of submassive PE 43,44. This condition is associated with a particularly high event mortality of approximately 30% and early identification of patients and hospitalization for treatment are imperative. In low-risk or nonmassive PE, systolic blood pressure remains normal and the markers that define massive or submassive PE are absent 42,43. These low-risk patients may be appropriate for ambulatory management if conditions allow.

Overall, PE is associated with a 90-day mortality of approximately 10–15% 44,45. Suspected PE should be considered a matter of clinical urgency due to the high mortality and morbidity associated with this condition. In an epidemiological model of fatal PE, 34% of affected patients experienced sudden death, 59% were undiagnosed during life and treated as having cardiac insufficiency or pneumonia and only 7% were correctly diagnosed with PE before death 47. Therefore, a large number of patients with PE may be misdiagnosed and would benefit significantly from a reliable diagnostic algorithm, particularly in countries where awareness of the disease is low.

### Diagnosing VTE in primary care

Data from the International Society on Thrombosis and Haemostasis “World Thrombosis Day” survey for Argentina, the only Latin American country included in the survey, indicate that the majority of the Argentinian population has little awareness or knowledge of DVT, PE, or their consequences, consistent with the results for all other countries evaluated in the survey 21,22. Consequently, the ability of general practitioners to diagnose this condition is critical.

**Figure 1:**
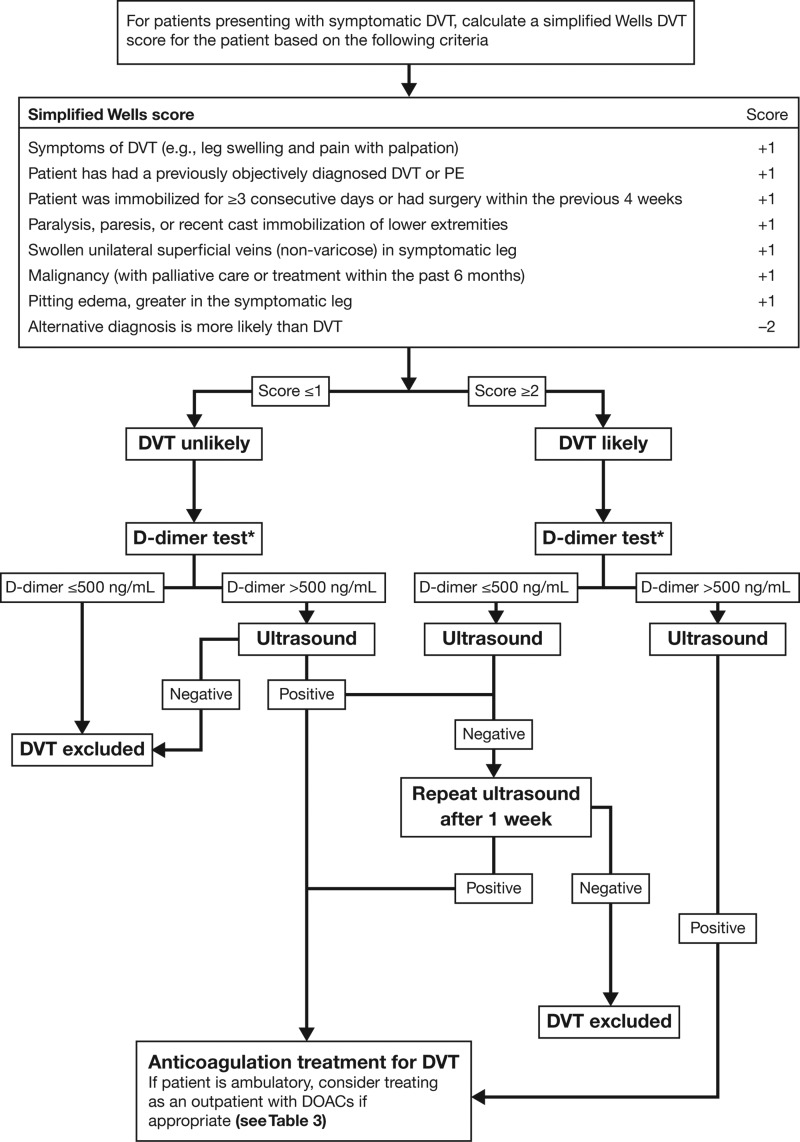
Diagnosis and treatment pathway for a patient presenting with symptomatic DVT. DOAC, direct oral anticoagulant (e.g., rivaroxaban, apixaban, dabigatran, or edoxaban); DVT, deep vein thrombosis; PE, pulmonary embolism. *Note: D-dimer cut-off should be age adjusted (age × 10 µg/L) in patients >50 years of age. Figure adapted from information in 35, 53, and 92.

In managing patients presenting with symptoms of DVT, such as a unilateral pitting edema or swollen and painful leg, or where there is clinical suspicion of DVT, the Wells DVT score or simplified Wells for predicting probability of DVT should be calculated before imaging is performed 48. If the score indicates a high probability of DVT, in the absence of contraindications, anticoagulation with heparin or similar agents should be initiated immediately while confirmatory tests are performed. However, if the score indicates a low probability of DVT, a negative D-dimer test can rule out a diagnosis of DVT without requiring imaging, although the D-dimer cut-off value used should be age-adjusted for older patients (age × 10 µg/L for patients >50 years of age). When screening a low-risk patient, treatment may be delayed only if the tests will be available within a reasonable time frame 31. A diagnostic algorithm for patients with DVT is presented in Figure 1 35.

In patients presenting with symptoms of PE, such as hemoptysis, chest pain and shortness of breath, or where there is clinical suspicion of PE, the Wells PE score 49 or revised Geneva score 50 should be calculated and used to predict the probability of PE before imaging is performed. As in DVT, for patients for whom there is a strong clinical suspicion of PE but no firm diagnosis, unless there are contraindications to heparin use, anticoagulation with unfractionated heparin, low-molecular-weight heparin (LMWH), or fondaparinux should be maintained until the diagnosis is confirmed 31. Bivalirudin or fondaparinux may be considered for patients who cannot receive heparin derivatives (e.g., as a result of heparin-induced thrombocytopenia) 31. The Pulmonary Embolism Severity Index and Simplified Pulmonary Embolism Severity Index (Table 1) can be used to assess the severity of the event based on 30-day survival probability 51,52.

**Figure 2:**
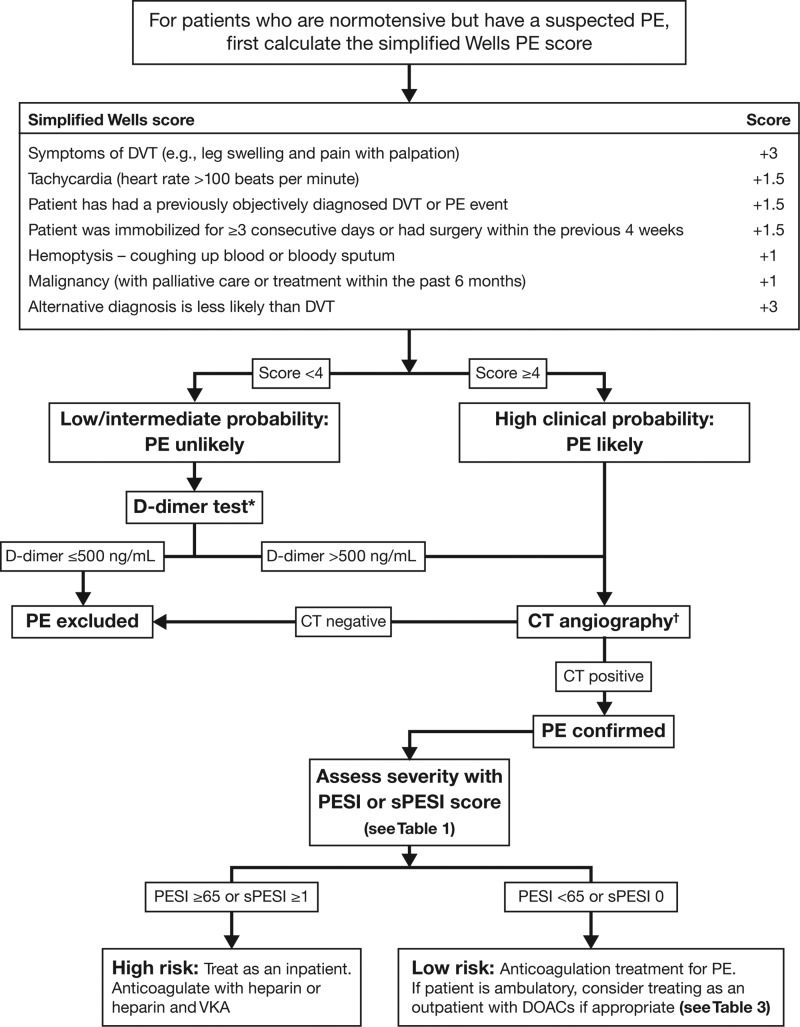
Diagnosis and treatment pathway for a patient presenting with symptomatic PE who is normotensive. CT, computed tomography; DOAC, direct oral anticoagulant (e.g., rivaroxaban, apixaban, dabigatran or edoxaban); DVT, deep vein thrombosis; PE, pulmonary embolism; PESI, Pulmonary Embolism Severity Index; sPESI, simplified PESI; VKA, vitamin K antagonist. *D-dimer cut-off should be age adjusted (age × 10 µg/L) in patients >50 years of age. ^†^For patients with renal failure, allergy to contrast dye, pregnant patients, or other contraindications to CT, a positive lower limb compression ultrasonography finding is sufficient to warrant anticoagulation. Figure 2 adapted from information in 53, 49, and 93.

A diagnostic algorithm based on the European Society of Cardiology guidelines for patients with suspected PE 52 in the absence of hypotension and shock (i.e., normotensive) is shown in Figure 2. For patients with suspected PE with suspected shock or hypertension, the European Society of Cardiology guidelines recommend that an angiography/helical CT should be performed if available, and treatment for PE initiated for positive findings 52. If the CT findings are negative, alternative causes for hemodynamic instability should be explored 52. If CT angiography is not immediately available, echocardiography should be performed to check for right ventricle (RV) overload and, if confirmed, the patient should be stabilized and then sent for CT 52. If the patient has confirmed RV overload, but cannot be stabilized or CT is not immediately available, treatment should be initiated for PE 52. In the absence of RV overload, alternative causes of hemodynamic stability should be investigated 52.

### Treatment options: guidelines, anticoagulant choices and treatment duration

The guidelines for individual countries in Latin America are generally consistent with those of the American College of Chest Physicians 23-25,31,. Guidelines on antithrombotic therapy for VTE recommend an anticoagulant for the treatment of acute VTE 31 to prevent further growth of the thrombus rather than to deplete the existing thrombus, for which fibrinolysis may be required. After the acute phase of treatment, the treatment pathways for DVT and PE are similar 31,54, reflecting that these are both manifestations of the same disease. The recommended duration of anticoagulation varies, depending on the cause of the initial event. Anticoagulation for 3 months is appropriate when there is a transient and reversible cause, such as surgery, trauma, or hospitalization; 6 months or more is appropriate for patients experiencing a first event of idiopathic VTE; and 12 months or more (possibly indefinitely, subject to regular review) is appropriate for patients with active neoplasia, recurrent idiopathic VTE, high-risk thrombophilia, or antiphospholipid antibodies 25. However, anticoagulation may be discontinued after the initial 3 months of treatment if the risk of bleeding is high 25. Due to the lower risk of recurrence associated with UEDVT, in the absence of additional factors, 3 months of anticoagulation treatment may be sufficient, even in cases of spontaneous DVT.

Initiation of parenteral anticoagulation is recommended without delay in patients with a high or intermediate probability of PE while diagnostic work-up is in progress. Heparin, LMWH, and fondaparinux are the recommended forms of parenteral anticoagulation in the acute phase 25,31,53. In parallel with parenteral anticoagulation, treatment with a vitamin K antagonist (VKA) is recommended, with a target international normalized ratio (INR) of 2.0-3.0. For inpatients with PE who have a high risk of bleeding or who are hemodynamically unstable and for whom precise anticoagulation control and the ability to quickly reverse anticoagulation may be desirable, unfractionated heparin may be preferred to LMWH due to its short half-life and the availability of an antidote, protamine 31.

Outpatient treatment is often appropriate for patients with low risk of acute DVT 54. A recent publication describes a very low rate of complications in Argentina in this setting, with only one major bleed and no recurrences in 359 ambulatory patients with DVT 55. VKAs can be effective for anticoagulation but have a slow onset/offset of action and require regular monitoring, which may be a barrier to their use, particularly in rural areas where access to anticoagulation specialists may be limited. When VKAs are used without careful monitoring and adjustment, there is an increased risk that patients will be outside the target therapeutic range (TTR) for longer periods, which is associated with an increased risk of both DVT recurrence and bleeding 56. Data from Latin America have included TTR values as low as 44% for atrial fibrillation patients in some locations 57. Results from international phase III studies in patients with atrial fibrillation, such as the Randomized Evaluation of Long Term Anticoagulant Therapy (RE-LY) trial, identified some Latin American countries in which the mean TTR was as low as 49% compared with the study average of 64% 58. A low TTR in this region has also been recorded in clinical trials, which have the advantages of patient selection according to trial criteria and close support and monitoring. Real-world TTR levels are likely to be at least 10-20% lower 59. This low rate of “in range” therapeutic anticoagulation could be a major disadvantage in Latin America and may be one of the region's most important challenges with respect to oral anticoagulation with VKAs. However, a recent study exploring TTR across 14 anticoagulation clinics in Argentina that included 1,190 consecutive patients with atrial fibrillation observed a mean TTR of 66.6%, a value similar to that observed in international therapeutic clinical trials and in Nordic countries, where care is largely available from highly developed socialized health systems, indicating that high-quality anticoagulation with VKAs is possible in Latin America 60.

### Direct oral anticoagulants

When the American College of Chest Physicians 2012 guidelines for the treatment of VTE were issued, there was insufficient clinical experience with direct oral anticoagulants (DOACs) to support recommendations for these agents over VKAs or LMWH 31. However, publications and clinical data from real-world use of DOACs have since become available and recommendations for their use were included in the more recent European Society of Cardiology guidelines 53. These new drugs are approved or under consideration for this indication by various regulatory agencies. DOACs offer rapid onset of action and predictable pharmacokinetics, obviating the need for regular monitoring in routine clinical use and enabling convenient oral administration. These qualities are significant advantages for DOACs compared with VKAs, and thus DOACs are particularly useful for outpatient treatment.

Ambulatory patients and those without cancer requiring long-term treatment for VTE may be good candidates for new oral anticoagulants if they have a low risk of bleeding and adequate renal and hepatic function. Several international experts have proposed that DOACs should be considered for patients in whom adequate anticoagulation with VKAs cannot be maintained in the therapeutic range despite good adherence to treatment and regular monitoring 61. Conversely, patients with poor adherence to treatment, patients with an underlying disease that requires hospitalization, or patients requiring triple antithrombotic therapy (e.g., dual antiplatelet therapy plus anticoagulant) may not be candidates for DVT treatment with DOACs. In addition, due to the relative scarcity of clinical experience, patients with cancer and thrombosis, antiphospholipid syndrome, high-risk thrombophilia, or thrombosis at an unusual site (e.g., splanchnic or cerebral vein thrombosis) should not be considered for DOACs until clinical trials have demonstrated the utility of these agents for these indications. In patients with severe renal impairment (creatinine clearance <30 mL/min), hepatic impairment (Child-Pugh category B or C), pulmonary thromboembolism with high burden of disease or a high-risk simplified Pulmonary Embolism Severity Index score (Table 1), severe hypotension, dilated right ventricle, or phlegmasia alba dolens (milk leg) (for the potential use of fibrinolytics), patients who are pregnant or breastfeeding and pediatric patients, traditional anticoagulant treatment either with heparins alone or in combination with VKAs may be a superior option 62,63.

The results of key studies of each of the DOACs are summarized in Table 2 64-71. In light of these clinical trial results, it will be important to reconsider treatment guidelines in some patients as new agents become available. Xarelto® (rivaroxaban) is already approved for the treatment of VTE/PE in Argentina, Brazil, Chile, Colombia, Mexico and Peru. Pradaxa® (dabigatran) is approved in Argentina, Brazil, Chile, Colombia and Mexico, and Eliquis® (apixaban) is currently approved in Argentina, Chile, Colombia, Mexico and Peru for this indication. Approval for all 3 agents in additional countries is expected in the near future. Guidelines for the use of rivaroxaban, apixaban, or dabigatran in the treatment of DVT or PE in ambulatory patients are presented in Table 3. At the time of writing, Savaysa™ (edoxaban) has not been approved for this indication in any Latin American country or in Europe and has yet to be included in the guidelines. However, prescribing information from the United States, where edoxaban was recently approved for the treatment of DVT and PE, states that edoxaban (60 mg once daily for patients with creatinine clearance >50 to ≤95 mL/min, reduced to 30 mg once daily in patients with creatinine clearance 15–50 mL/min, who weigh ≤60 kg, or who are taking specific concomitant P-glycoprotein inhibitor medications) can be used following 5 to 10 days of initial therapy with a parenteral anticoagulant 72.

### Barriers to widespread direct oral anticoagulant use

Access to the healthcare system can present a significant barrier to care in general, with WHO estimates indicating that more than 40% of the population of some countries does not have effective access to healthcare for reasons ranging from language barriers and a lack of education to a lack of health infrastructure and lack of access to other public services, including electricity and sanitation, which thus impairs adequate provision of healthcare 73. Where access to the healthcare system is available, one of the major barriers to appropriate anticoagulation in Latin America is economic; the high pharmacy cost of outpatient anticoagulation treatment can make it inaccessible to patients who must pay drug costs out of pocket 73 and when funded publicly, the use of more expensive drugs may be restricted or controlled centrally 13. Consequently, ambulatory patients who would be suitable for outpatient treatment with new oral anticoagulants may be admitted to the hospital to receive the first few days of treatment as inpatients. In some institutions, patients may be admitted to receive unfractionated heparin via continuous infusion pump to avoid the high cost of LMWH 13,26, despite the greater overall cost to the healthcare system of doing so 74,75. Thus, while it may be theoretically possible for 80% of patients with DVT to be managed on an outpatient basis 76,77, the actual rate of outpatient care is much lower and both institutional and healthcare system costs are increased.

Across many parts of Latin America there is a lack of knowledge regarding the pathology of VTE, leading to late consultation. Furthermore, even if a clinician has adequate evidence to suspect a diagnosis requiring anticoagulation, therapy is occasionally not started until the diagnosis has been confirmed, which in some institutions results in an additional delay of several days while waiting for confirmation by ultrasound.

In some regions of Latin America, particularly rural areas, access to an anticoagulation clinic may be difficult. Therefore, in many countries, patients may be managed by general physicians (rather than anticoagulation specialists), who may lack detailed knowledge of how to evaluate anticoagulated patients. Non-specialists are also less likely to be aware of the range of available treatments and when oral anticoagulation is prescribed, may choose traditional anticoagulation, such as a VKA, based on familiarity, despite the additional burden of monitoring.

### Emergency reversal of anticoagulant effects

Evidence suggests that 4-factor prothrombin complex concentrates may be as useful for managing bleeding related to factor Xa (FXa) inhibitors and direct thrombin inhibitors as they are for bleeding related to VKAs 78-81; however, the use of DOACs has been limited by the lack of a specific active reversal agent or antidote for use in bleeding management or prior to an emergency procedure. Idarucizumab (Praxbind), an antibody fragment–based reversal agent for dabigatran, was approved in late 2015 82 and results from phase III studies of andexanet alfa, a recombinant modified FXa for the reversal of FXa inhibitors, are positive 83. Also under development is PER977, a synthetic small molecule that may eventually become a wide-range reversal agent for anticoagulants. PER977 exhibits complete reversal of FXa inhibition, direct thrombin inhibition, and the anticoagulant effects of LMWH, unfractionated heparin, and fondaparinux both in vitro and in preclinical animal models and, more recently, for the reversal of edoxaban, a FXa inhibitor, in healthy subjects 84.

### Monitoring of anticoagulant effect

The use of DOACs has also been limited by the lack of a readily available standardized assay for anticoagulant activity. Despite the lack of a requirement for routine monitoring with DOAC administration, situations may arise where monitoring is desirable. The standard clotting assays used with heparin derivatives and VKAs cannot be used to quantitatively assess anticoagulant activity with DOACs, but other assays are becoming available to facilitate point-of-care testing. A thrombin inhibitor assay suitable for use with dabigatran is now commercially available, although its real value in the clinical setting remains controversial 85. Tests based on chromogenic FXa-specific inhibitor assays with appropriate calibrators are now commercially available to assess the anticoagulant activity of anti-FXa drugs such as rivaroxaban and apixaban, but their clinical relevance has yet to be evaluated 86,87.

### Adherence to treatment

Although the lack of a requirement to routinely monitor anticoagulation with DOACs may be advantageous in some situations, it may actually increase the likelihood of patients stopping their anticoagulant treatment earlier than recommended. Adherence may be particularly problematic where health literacy is relatively low, such as in some Latin American regions, and thorough education is needed to ensure that patients adhere to their treatment regimens as prescribed.

### Increased drug costs

Drug purchase costs for DOACs are significantly greater than those for generic warfarin. Cost is an important limitation to the use of DOACs in Latin America, where many patients incur high out-of-pocket health costs, with estimates for the average percentage of household income spent on healthcare varying by country from 2.0% to 6.9% 73. At present, the increased drug cost may be partially offset by a reduction in the costs associated with regular monitoring of the anticoagulant effect, as well as potentially reduced costs relating to bleeding events and hospitalizations. In addition, as DOACs become more widely used, competition among the different agents may result in lower prices over time. The higher drug costs associated with DOACs compared with VKA therapy could be a factor in a patient's decision to discontinue anticoagulant therapy earlier than would be advised by a healthcare provider.

### Clinical experience

Finally, a lack of clinical experience in a specific patient population compared with standard anticoagulant therapies may also be a barrier to treatment. Clinical trial populations may not accurately reflect the patient population in real-world clinics, who are subject to multiple comorbidities 78, polypharmacy 79 and other potential modifiers that would have resulted in the exclusion of these patients from the original trials. In addition to comorbidities and polypharmacy associated with an increased risk of bleeding and worse clinical outcomes 79-81, the presence of additional risk factors for bleeding may make physicians more cautious about prescribing anticoagulants in general, particularly those with which they have limited clinical experience. Over time, more data regarding DOAC use in real-world patient populations will become available, and as clinical experience with these agents increases, this barrier to DOAC use is likely to be lowered.

VTE is a significant health problem in Latin America and is complicated by various region-specific issues. In some regions, awareness of diagnostic criteria for VTE is low. Many patients do not receive appropriate anticoagulation even after being diagnosed with VTE/PE. Clear guidance to facilitate the diagnosis of VTE and provide appropriate anticoagulation for patients once diagnosed may offer significant benefits in the region. Heparin-based anticoagulants are likely to remain the first choice for inpatients with VTE at high risk of bleeding or with additional complications for which the doctor may desire a precise level of control over anticoagulation. However, DOACs may be particularly beneficial for outpatients requiring anticoagulation, particularly in situations in which regular monitoring may not be feasible.

#### FINANCIAL SUPPORT

Dr. Ceresetto is on the Advisory Board for Bristol-Myers Squibb in Argentina.

#### SPONSORSHIP

Professional medical writing and editorial assistance were provided by Andy Shepherd and Nicole Draghi at Caudex Medical, funded by Bristol-Myers Squibb Company and Pfizer Inc. Dr. Ceresetto is on the Advisory Board for Bristol-Myers Squibb in Argentina and has received support for this manuscript from Bristol-Myers Squibb Company and Pfizer Inc.

## Figures and Tables

**Table 1:** PESI and sPESI: prognostic scores for severity in patients with acute symptomatic pulmonary embolism.

PESI Score		Simplified PESI Score	
Variable	Points	Variable	Points
Age	1/year	Age >80 years	1
Male sex	10	Cancer	1
Cancer	30	Chronic cardiopulmonary disease	1
Heart failure	10	Heart rate ≥110 beats/min	1
Chronic lung disease	10	Systolic blood pressure <100 mm Hg	1
Heart rate ≥110 beats/min	20	O_2_ saturation <90%	1
Systolic blood pressure <100 mm Hg	30		
Respiratory rate ≥30 breaths/min	20		
Temperature <36°C	20		
Altered mental status	60		
O_2_ saturation <90%	20		
***Interpretation*:** Class I (very low risk): <65 points Class II (low risk): 66-85 points Class III (intermediate risk): 86-105 points Class IV (high risk): 106-125 points Class V (very high risk): >125 points		***Interpretation*:**Low risk: 0 pointsHigh risk: ≥1 point(s)	

PESI: Pulmonary Embolism Severity Index; sPESI: simplified PESI.

Table adapted from 49 and 55.

**Table 2:** Summary of key clinical trials of novel oral anticoagulants in the treatment of venous thromboembolism and pulmonary embolism.

Study	Patient population	Study treatment	Key findings
**Dabigatran**			
RE-COVER/ RE-COVER II (64) (66)	Patients with acute symptomatic VTE	Heparin or LMWH for 8-11 days, followed by dabigatran 150 mg BID or warfarin given in doses adjusted to an INR of 2.0-3.0 for 6 months	Dabigatran was noninferior to warfarin for the prevention of recurrent or fatal VTE and had a similar rate of major bleeding but a lower risk of any bleeding events.
			
RE-MEDY (65)	Patients completing at least 3 months of VTE treatment who were considered at increased risk of recurrent VTE	Dabigatran 150 mg or warfarin (adjusted to an INR of 2.0-3.0)	Dabigatran was noninferior to warfarin (adjusted to an INR of 2.0-3.0) for the prevention of recurrent symptomatic and objectively verified VTE or death associated with VTE.
			
RE-SONATE (65)	Patients completing at least 6 months of treatment for VTE.	Dabigatran 150 mg BID or placebo	Dabigatran significantly reduced the rate of recurrent VTE but with a significantly higher rate of major or CRNM bleeding (5.3% *vs*. 1.8%).
**Rivaroxaban**			
EINSTEIN-DVT (67)	Patients with acute symptomatic DVT in the deep veins of the knee or thigh, but without any symptoms of PE	Rivaroxaban or standard therapy (enoxaparin followed by VKA) for 3, 6, or 12 months	Rivaroxaban had non-inferior efficacy with respect to the primary outcome, with similar rates of major and CRNM bleeding.
			
EINSTEIN-PE (68)	Patients with acute symptomatic PE with or without symptomatic DVT	Rivaroxaban (15 mg BID for 3 weeks, followed by 20 mg QD) or standard therapy (enoxaparin followed by VKA) for 3, 6, or 12 months	Rivaroxaban was non-inferior to enoxaparin/VKA therapy for the prevention of recurrent VTE, with similar rates of total and CRNM bleeding. However, rivaroxaban was associated with a statistically significant reduction in major bleeding events (HR, 0,49 [0,31-0,79]) in patients with PE.
			
EINSTEIN-EXT (67)	Patients who had previously completed 6 to 12 months of treatment with a VKA for an acute episode of VTE or had participated in the EINSTEIN-DVT or EINSTEIN-PE trials	Rivaroxaban 20 mg QD or placebo for 6 to 12 months	Rivaroxaban demonstrated superiority to placebo for the primary outcome. Efficacy and safety results were consistent across all prespecified subgroups.
**Apixaban**			
AMPLIFY (69)	Patients presenting with acute DVT or PE	Apixaban 10 mg BID for 7 days followed by 5 mg BID for 6 months or subcutaneous enoxaparin for 5 days followed by dose-adjusted warfarin for 6 months	There were no significant differences in the rates of the primary efficacy outcome of recurrent symptomatic VTE or VTE-related death (2.3% *vs*. 2.7%, respectively) between treatments, and apixaban was associated with significantly fewer major bleeding and CRNM bleeding events compared with conventional therapy (events occurred in 4.3% *vs*. 9.7% of patients, respectively).
			
AMPLIFY-EXT (70)	Patients with DVT/PE for whom there was clinical uncertainty about whether to continue oral anticoagulation after 6-12 months of routine treatment with a VKA	Placebo, apixaban 2.5 mg BID, or apixaban 5 mg BID for 12 months	Compared with placebo, both doses of apixaban reduced the risk of recurrent fatal or nonfatal VTE, while rates of major bleeding were low and comparable to those in the placebo group.
**Edoxaban**			
HOKUSAI-VTE (71)	Patients who presented with DVT or PE	5-7 days of heparin followed by edoxaban 30 or 60 mg QD or warfarin for 3-12 months	Edoxaban was noninferior to warfarin with respect to the primary efficacy outcome of recurrent symptomatic VTE, with less major or CRNM bleeding compared with the warfarin group.

BID: twice daily; CRNM: clinically relevant non-major; DVT: deep vein thrombosis; HR: hazard ratio; INR: international normalized ratio; LMWH: low-molecular-weight heparin; PE: pulmonary embolism; QD: once daily; VKA: vitamin K antagonist; VTE: venous thromboembolism.

**Table 3:** Anticoagulation therapy for patients with DVT or patients with PE in the absence of hypotension or shock.

Treatment	Acute phase	Maintenance phase
**VKA + parenteral anticoagulation**	7-10 days of treatment with LMWH, UFH, or fondaparinux (as appropriate)	VKA with a target INR of 2.0-3.0 to be initiated in parallel to parenteral anticoagulation
		
**Rivaroxaban** can be considered as an alternative to the combination of parenteral anticoagulation and VKA in low-risk or ambulatory patients.	15 mg BID taken with food for 3 weeks	20 mg QD taken with food, initiated immediately following acute phase treatment
**Apixaban** can be considered as an alternative to the combination of parenteral anticoagulation and VKA in low-risk or ambulatory patients.	10 mg BID for 7 days	5 mg BID, initiated immediately following acute phase treatment
**Dabigatran** can be considered as an alternative to VKA in ambulatory patients following 7-10 days of treatment with a parenteral anticoagulant.	**Not approved**–use parenteral anticoagulation	150 mg BID or 110 mg BID in patients ≥80 years of age, initiated immediately following acute phase treatment

See prescribing information for individual agents for further information on contraindications or dosage adjustments in certain patient groups. All recommendations below are subject to local regulatory approval of these agents for this indication.

BID: twice daily; DVT: deep vein thrombosis; INR: international normalized ratio; LMWH: low-molecular-weight heparin; PE: pulmonary embolism; QD: once daily; UFH: unfractionated heparin; VKA: vitamin K antagonist.
